# Exosomal lncRNA PCAT-1 promotes Kras-associated chemoresistance via immunosuppressive miR-182/miR-217 signaling and p27/CDK6 regulation

**DOI:** 10.18632/oncotarget.27675

**Published:** 2020-07-21

**Authors:** Kalliopi Domvri, Savvas Petanidis, Doxakis Anestakis, Konstantinos Porpodis, Chong Bai, Paul Zarogoulidis, Lutz Freitag, Wolfgang Hohenforst-Schmidt, Theodora Katopodi

**Affiliations:** ^1^Pulmonary Department-Oncology Unit, “G. Papanikolaou” General Hospital, Aristotle University of Thessaloniki, Thessaloniki, 57010, Greece; ^2^Department of Medicine, Laboratory of Medical Biology and Genetics, Aristotle University of Thessaloniki, Thessaloniki, 54124, Greece; ^3^Department of Pulmonology, I.M. Sechenov First Moscow State Medical University, Moscow, 119992, Russian Federation; ^4^Department of Medicine, Laboratory of Forensic Medicine and Toxicology, Aristotle University of Thessaloniki, Thessaloniki, 54124, Greece; ^5^Department of Respiratory & Critical Care Medicine, Changhai Hospital, Second Military Medical University, Shanghai, 200433, China; ^6^Third Department of Surgery, AHEPA University Hospital, Aristotle University of Thessaloniki, Thessaloniki, 55236, Greece; ^7^Department of Pulmonology, University Hospital Zurich, Rämistrasse 100, 8091, Zurich; ^8^Medical Clinic I, Fuerth Hospital, University of Erlangen, Fuerth, 91054, Germany

**Keywords:** PCAT-1, miR-182, immunosuppression, Kras, miR-217

## Abstract

Immunosuppressive chemoresistance is a major burden in lung cancer. Recent data reveal that long noncoding RNAs (lncRNAs) present in the lung tumor microenvironment are implicated in chemoresistant-related immune deregulation, and metastasis but their exact pathogenic role is still unknown. In this study, we investigate the role of lncRNA PCAT-1 in chemoresistant immunosuppression and its involvement in tumor stroma remodeling. Findings reveal PCAT-1 to regulate Kras-related lung chemoresistance through increased expression of the immunosuppressive micrornas miR-182/miR217 in lung tissues, thus promoting a pre-metastatic niche formation and a subsequent increase in lung metastatic burden. Elevated expression of PCAT-1 negative regulates p27/CDK6 expression by inducing G_0_/G_1_ cell cycle arrest through AMPK augmentation, contributing to a tumor-promoting status. Furthermore, PCAT-1 triggered fibroblast differentiation followed by CAF/myofibroblast secretion in TME triggering a CD133/SOX2-related stem cell phenotype. Subsequent PCAT-1 knockdown impaired CAF-mediated stromal activation, and reversed chemoresistance and tumor growth *in vivo*. Overall, these findings demonstrate the versatile roles of PCAT-1 in sustaining lung immunosuppressive neoplasia through tumor microenvironment remodeling and provide new opportunities for effective metastasis inhibition, especially in chemoresistant tumors.

## INTRODUCTION

Lung cancer remains the leading cause of cancer-related deaths and despite extensive research efforts, the survival rate of lung cancer patients remains significantly low [1, 2]. Even though new immunotherapy methods and improved surgical techniques have been introduced, lung cancer remains a significant therapeutic challenge [3–5]. Emerging evidence indicates that lncRNAs present in the lung tumor microenvironment promote tumor growth through cancer cell remodeling that favors immunosuppressive metastasis [6, 7]. These decisive tumor propagating effects of lncRNA permit tumor cells to bypass immune surveillance and reduce T-cell infiltration into tumor, limiting the clinical benefits of immune checkpoint therapies [8–10].

LncRNAs are a type of noncoding RNAs longer than 200 nucleotides in length which are involved in several cellular process including cell division, metabolism, proliferation, apoptosis, and differentiation [11–13]. Recent reports show that lncRNAs can act as oncogenes or tumor suppressors in malignant tumors through transcriptional regulation of gene expression, recruitment of chromatin-remodeling complexes and interaction with miRNAs or mRNAs [14, 15]. Increasing evidence reveals that intratumoral lncRNA levels correlate with tumor progression and metastasis through tumor microenvironment remodeling [16, 17].

Fibroblasts which play a key role in this mechanism, constitute most of the stromal cells in tumor tissues, secrete a wide spectrum of chemokines or cytokines to the tumor microenvironment, thus promoting growth, invasion, angiogenesis [18, 19]. Specifically lncRNAs are released by exospores that can alter this fibroblast phenotype and shift their role from being immunostimulatory to immunosuppressive at various stages of carcinogenesis [20, 21]. Tumor cells or immune cells can also release exosomal factors that inhibit or reverse fibroblast maturation and alter their normal function [22]. Especially, fibroblasts can be “educated” by lung cancer cells to mediate immunosuppression and promote tumor growth by stimulating angiogenesis [23, 24]. Recent reports describe the presence of tumor promoting microRNAs which have overlapping associations with fibroblast cell function and immune response regulation [25, 26]. Immune deregulation in the tumor microenvironment plays a key role in patients survival. Especially, the deregulation of miRNAs and exosomal miRNAs can influence fibroblast differentiation towards a CAF phenotype in the tumor microenvironment [27, 28]. CAFs (cancer-associated fibroblasts) are the major components of the tumor microenvironment. Tumor cells appear to transform normal-associated fibroblasts (NAFs) into CAFs involving direct cell-cell communication and epigenetic regulations [29]. Furthermore, fibroblast-derived exosomes induce cancer stem cell expression that contributes to altered tumor metabolism and emergence of chemoresistance in tumor microenvironment [30, 31].

In this study we characterize for the first time the role of lncRNA PCAT-1 in Kras-related lung chemoresistance and its role in tumor stroma remodeling via immunosuppressive miR-182/miR217 expression and fibroblast differentiation. Aberrant expression of PCAT-1 in the tumor microenvironment negative regulates p27/CDK6 by inducing G_0_/G_1_ cell cycle arrest and AMPK augmentation, contributing to a tumor-favoring metabolic status. Our findings highlight the crucial relationship between CAFs and PCAT-1 which triggers a CD133/SOX2-related stem cell phenotype and acquisition of cancer cell chemoresistance. PCAT-1 knockdown impaired CAF-mediated stromal fibroblast activation, and tumor-related chemoresistance mechanism through induction of T cell recruitment and activation of IL-13/IL-33-mediated pathway. Overall, these findings demonstrate the multiple roles of PCAT-1 in sustaining lung immunosuppressive metabolism and provide new opportunities for effective inhibition of metastatic lung cancer.

## RESULTS

### Mutant Kras-related chemoresistance is associated with increased expression of lncRNA PCAT-1 in metastatic lung cancer

**Figure 1:**
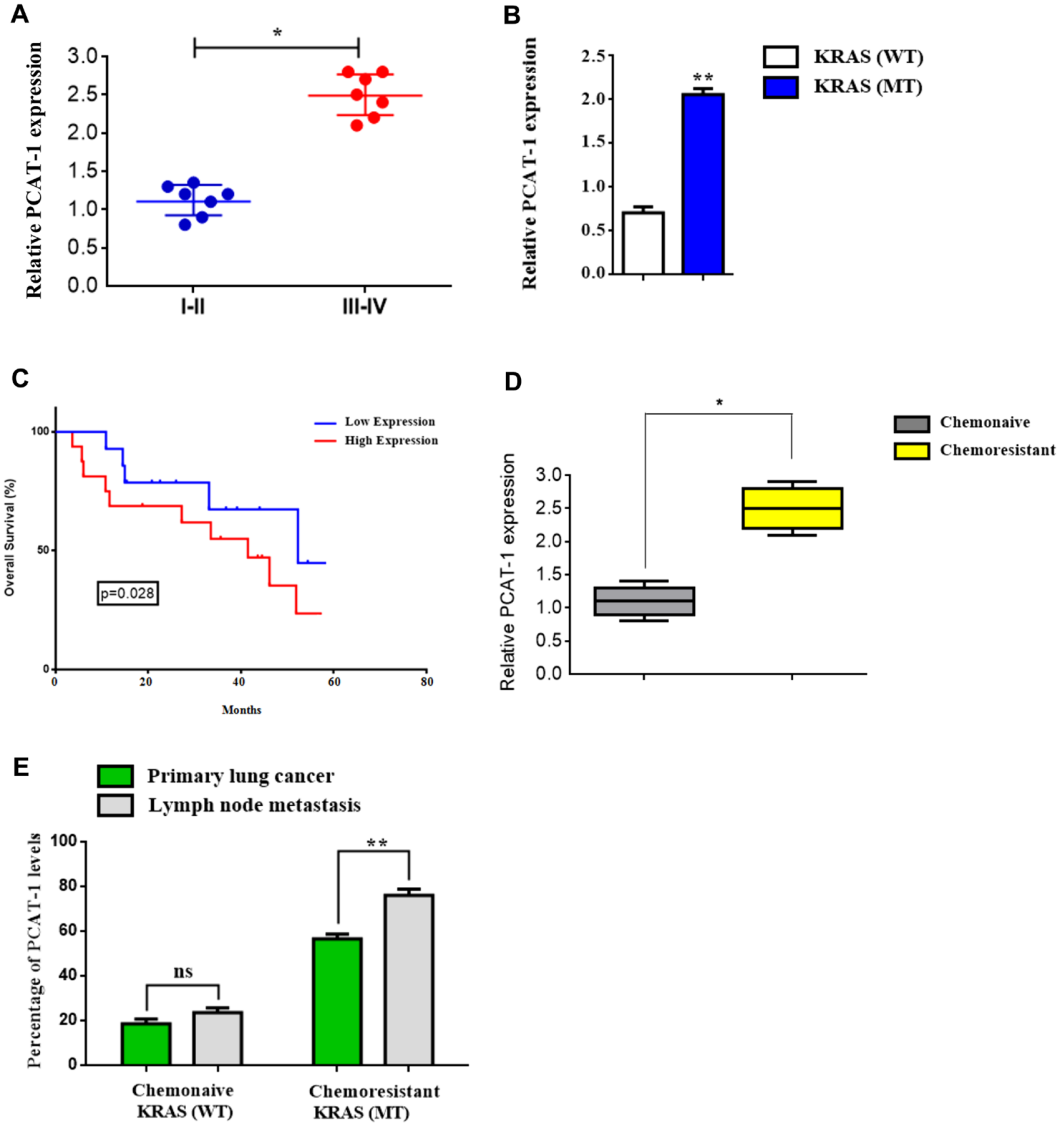
Mutant Kras-related chemoresistance is associated with increased expression of lncRNA PCAT-1 in metastatic lung cancer. (**A**) Relative PCAT-1 expression levels in early stage (I-II) lung cancer and late metastatic (III-IV) tumor stage using RT-PCR. (**B**) Relative expression of PCAT-1 in Kras wild type (WT) and mutant (MT) lung cancer patients. (**C**) Kaplan-Meier curves of the survival rate of lung cancer patients in groups of PCAT-1 high or low expression levels. (**D**) Relative expression of PCAT-1 in chemoresistant or chemonaive cancer patients. (**E**) Percentage rates of PCAT-1 expression from primary lung cancer or lymph node metastasis biopsy tissues from Kras (WT/MT) patients. The results represent the mean ± SD of three independent experiments. Differences were considered statistically significant at *p* < 0.05. Statistically significant data are indicated by asterisks (^*^ *P* < 0.05, ^**^ *P* < 0.01).

Recent studies reveal that PCAT-1 plays a significant role in metastatic tumor progression and acquisition of chemoresistance. However, Kras chemoresistance-related expansion of heterogeneous lncRNA subsets in primary or metastatic sites and their molecular mechanism of interaction within the tumor microenvironment remains unknown. To address this issue, we investigated the expression of PCAT-1 by qRT-PCR in Kras (WT/MT) lung tumor and metastatic lymph node tissues. Findings reveal that expression of PCAT-1 was significantly upregulated in metastatic (Stage III–IV) tumor tissues compared with early stage neoplasia (I–II) tissues (Figure 1A). Furthermore, mutant (MT) Kras samples showed a higher expression of PCAT-1 in relation with wild type (WT) Kras tissues (Figure 1B, Supplementary Table 3). Further investigation of the clinical significance of PCAT-1 expression in patient overall survival (OS) analysis revealed that higher expression of PCAT-1 in LC patients was correlated with short OS (Figure 1C). Kaplan–Meier surviving curves were employed for the analysis of the prognostic potential of PCAT-1 in LC patients. Data show that increased levels of PCAT-1 were closely associated with poor patient prognosis (Figure 1C). Likewise, the relative highest levels of PCAT-1 were detected in chemoresistant patients in advanced stage rather than in chemonaive-responding to chemotherapy regimen- early stage individuals, indicating the critical role of PCAT-1 in tumor propagation (Figure 1D). Additionally, aberrant PCAT-1 expression was observed in chemoresistant Kras (MT) patients in both primary and lymph node metastasis regions in relation to chemonaive wild type Kras individuals (Figure 1E). Taken together, these findings indicate the crucial role of PCAT-1 in establishing Kras-related chemoresistance and its involvement in the metastatic niche.

### LncRNA PCAT-1 negative regulates p27/CDK6 by inducing G_0_/G_1_ cell cycle arrest through AMPK augmentation

**Figure 2:**
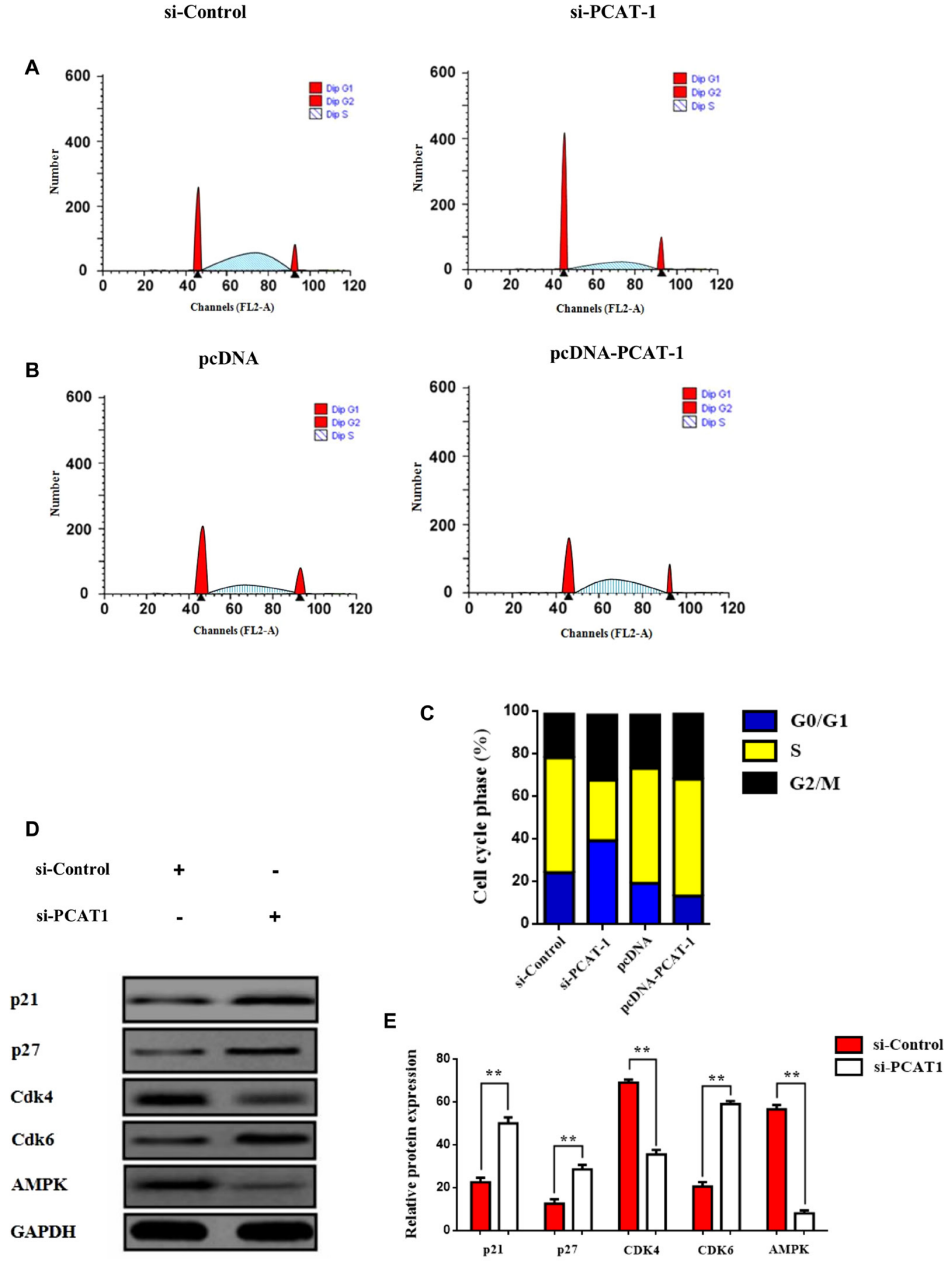
PCAT-1 negative regulates p27/CDK6 by inducing G_0_/G_1_ cell cycle arrest through AMPK amplification. (**A**–**B**) Flow cytometry assay of H1975 cells transfected with pcDNA-PCAT-1/pcDNA or si-PCAT-1/si-Control and then subjected to cell cycle analysis. The results represent the mean ± SD of three independent experiments. (**C**) Histogram showing each phase of the cell cycle following transfection of H1975 cells with pcDNA-PCAT-1/pcDNA or si-PCAT-1/si-Control. (**D**) The protein expression levels of p21, p27, cyclin D2, cdk4 and AMPK were assayed by Western blot following transfection of A549 cells with si-PCAT-1/si-Control. A549 cells were transfected with siRNA against PCAT-1 (4 μg) for 24 h. (**E**) Densitometric analysis of each protein level was calculated from the average of three experiments. Each value was expressed as the ratio of the measured protein to GAPDH level. Data represent the mean ± SD of three independent experiments (^*^ *P* < 0.05; ^**^ *P* < 0.01).

To further investigate whether the chemoresistance-related tumorigenic mechanism of PCAT-1 [32] is associated with metabolism regulation through cell cycle control, we performed cell cycle analysis using flow cytometry. Findings provide evidence that PCAT-1 can regulate tumor cell fate and cell differentiation, by disrupting cell cycle sequence. Moreover, results showed that PCAT-1 promoted G_0_/G_1_cell cycle arrest as shown by the increase of cells in the S phase following transfection with pcDNA-PCAT-1 plasmid (Figure 2A, Supplementary Figure 1). Augmented PCAT-1 expression enhanced the percentage of cells in S phase and decreased the G0/G1-phase percentage. Furthermore, G2/M and S cell population were significantly increased in pcDNA-PCAT-1 overexpressing cells (Figure 2B–2C), implying an important role of PCAT-1 in regulating the G1/S transition. Knockdown of PCAT-1 expression, restored G2/M and S cell cycle levels and rescue cell proliferation. Also, proteins involved in the cell cycle regulation were analyzed by Western blot, and results showed that PCAT-1 increased expression of AMPK/CDK4 whereas negative regulated the levels of p21, p27 and CDK6 respectively (Figure 2D–2E). Interestingly, recently deregulated CDK4/CDK6 levels were closely associated with immune suppression and control of cancer immune surveillance [33].

### PCAT-1 triggers the CD133/SOX2-related stem cell phenotype of metastatic tumor cells via myofibroblast activation

**Figure 3:**
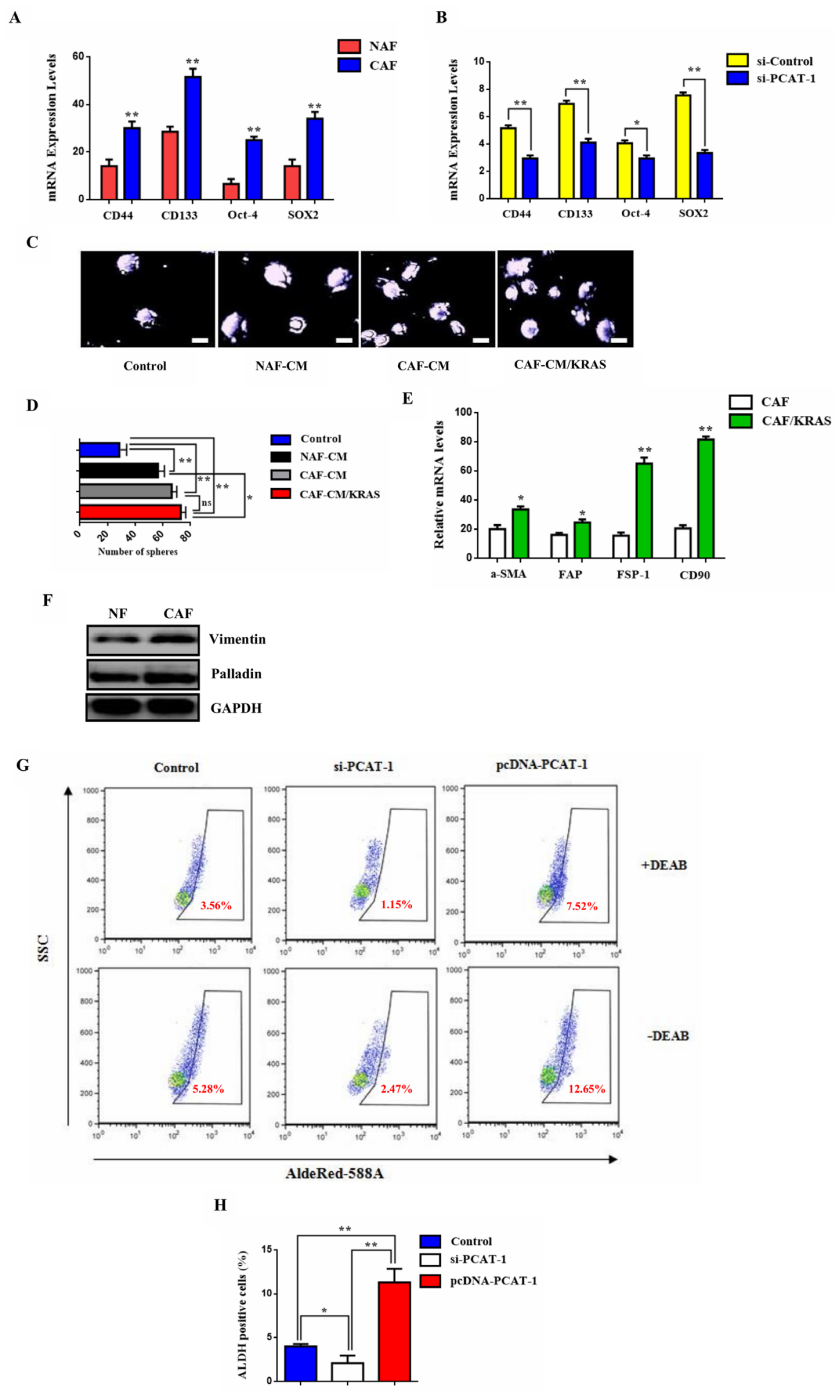
PCAT-1 activates the CD133/SOX2-related stem cell phenotype of metastatic tumor cells via myofibroblast differentiation. (**A**) qRT-PCR was employed to analyze the mRNA levels of the CSC-related genes CD44, CD133, OCT-4 and SOX2. (**B**) Effect of PCAT-1 knockdown in CSC gene expression. (**C**) Representative images from sphere formation assay following cell incubation with various culture mediums for 48 h. (**D**) Quantitative analysis of the sphere formation rates. Data represent the mean ± SD of three independent experiments. (**E**) RT-PCR analysis of the CAF-related genes a-SMA, FAP, FSP1, and CD90. (**F**) Western blot analysis of the myofibroblast biomarkers Vimentin and Palladin in NAF/CAF-treated cells. (**G**) AldeRed detection assay was performed to detect ALDH1^+^ cell populations. (**H**) Statistical analysis of the number of ALDH1^+^ cells in each group. Data represent the mean ± SD of three independent experiments (^*^ *P* < 0.05; ^**^ *P* < 0.01). For the RT-PCR GAPDH was used as an internal control in the mRNA analysis experiments.

Recent reports reveal that CAF can regulate lncRNA expression in tumor microenvironment and amplify their tumorigenicity [34, 35]. To assess this we treated lung tumor cells with conditioned medium (CM) derived from normal fibroblasts (NF) or CAFs and we investigated the role of self renewal mechanism in lung TME. Moreover, compared to the NF-treated cells, the expression of cancer stem cell (CSC) markers CD44, CD133, OCT-4 and SOX2, was significantly upregulated in the CF group (Figure 3A). Specifically, the high expression levels of CD133 and SOX2 play a crucial role in the acquisition of metastatic phenotype by tumor cells, and are also implicated in cancer cell differentiation [36]. Suppression of PCAT-1 inhibited the self-renewal acquisition mechanism in lung tumor cells, and it significantly reduced the expression of CSC markers (Figure 3B). Next, we investigated the sphere forming capacity of CAF/NF-treated cells in the presence or absence of KRAS. Data analysis reveal that KRAS overexpression in CAF medium enhanced the sphere-propagating capacity in lung cancer cells, in comparison with NF of CAF alone groups (Figure 3C–3D). To further characterize CAF phenotype the expression of fibroblast specific biomarkers was measured. RT-PCR analysis of CAF-related genes, including alpha-smooth muscle actin (a-SMA) fibroblast activation protein (FAP), fibroblast specific protein 1 (FSP1), and CD90, were distinctly unregulated compared to the expression in NFs (Figure 3E). Interestingly, both groups show myofibroblast activation as indicated by the myofibroblast biomarkers vimentin and paladin (Figure 3F). In particular, CAF-treated cells show a highly significant positive expression of these markers which indicates the correlation of myofibroblasts with disease progression and metastasis. As myofibroblasts have been reported to play a key role in immune suppression and chemoresistance we decided to monitor cell populations of ALDH1^+^ tumor which express cancer stem cell marker and correlate with poor prognosis. By using the AldeRed detection assay ALDH1 high cell population was augmented by PCAT-1 overexpression and was down-regulated in the siPCAT-1 knockdown group (Figure 3G–3H). These findings indicate the key role of PCAT-1 in sculpturing the stem cell phenotype of metastatic tumor cells via CAF/myofibroblast activation.

### Crosstalk of PCAT-1 with immunosuppressive miR182/miR-217 signaling axis

**Figure 4:**
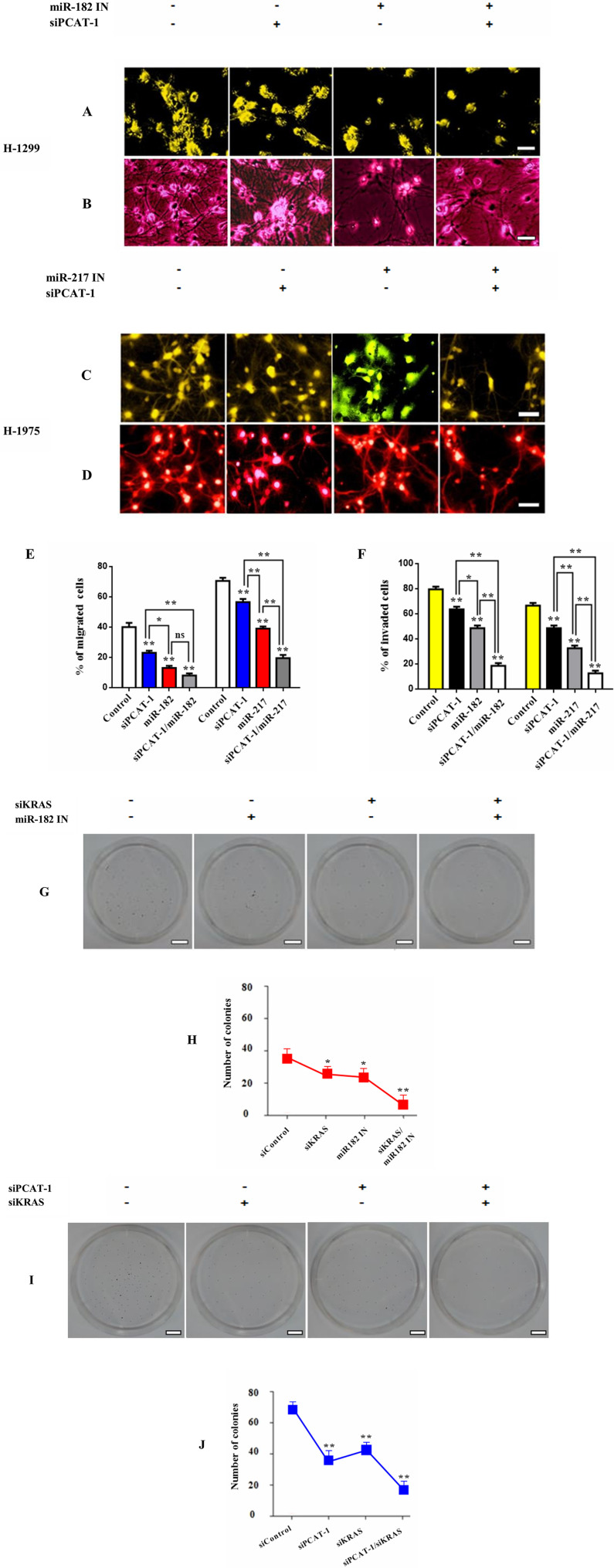
PCAT-1 regulates expression of immunosuppressive miR182/miR-217 signaling axis. (**A**–**B**) Representative images from migration and invasion assays of H1299 cells after transfection with siRNA against PCAT-1 (4 μg) and miR-182 inhibitor (25 μΜ) for 24 h. (**C**–**D**) Representative images from migration and invasion assays of H1975 cells after transfection with siRNA against PCAT-1 (4 μg) and miR-217 inhibitor (25 μΜ) for 24 h. (**E**–**F**) Quantitative analysis of migration and invasion assays following cell transfection. The results represent the mean ± SD of three independent experiments. (**G**–**H**) Colony formation assay of H1975 cells after transfection with siRNA against KRAS (4 μg) and miR-182 inhibitor (25 μΜ) for 24 h. (**I**–**J**) Colony formation assay of H1975 cells following co-transfection with siRNAs against KRAS (4 μg) and PCAT-1 (4 μg) for 24 h. Differences were considered statistically significant at *p* < 0.05. Statistically significant data are indicated by asterisks (^*^ *P* < 0.05, ^**^ *P* < 0.01).

Increasing evidence suggests that lncRNAs can act as ceRNAs and regulate the expression of specific miRNAs [37, 38]. To better comprehend this mechanism of PCAT-1 induced immunosuppression we investigated the role of known immunosuppressive miRs miR-182 and miR-217 in lung microenvironment remodeling. Metastatic H1299 and H1975 lung cells were treated with siPCAT-1 and miR-182/miR-217 inhibitors and subjected to invasion or migration analysis respectively (Figure 4A–4D). Co-treatment with siPCAT/miR-182 inhibitor significantly reduced the percentage of migrated cells compared with siPAT-1 or miR-182 inhibitor alone. (Figure 4C–4D). On the other hand, cells transfected with siPCAT-1/miR-217 inhibitor showed lower reduction in migration rates compared with miR-182 inhibitor group or single treatments. In addition, cell invasiveness was significantly restricted in the siPCAT-1/miR-217 group, whereas co-treatment with siPCAT/miR-182 inhibitor reduced the percentage of invaded in a lower rate (Figure 4E–4F). Since Kras plays a key role in affecting the tumorigenic profile of lncRNAs we decided to investigate the effect of Kras signaling in PCAT-1 and immunosuppressive microRNA signaling. To investigate this hypothesis we performed colony formation assay. Results demonstrate that co-inhibition of Kras/miR-182 downregulates colony formation rate significantly in relation to siKras/siPCAT-1 and control group treatment (Figure 4G–4J, Supplementary Figure 2). These finding designate the decisive role of Kras in establishing lung immunosuppression mainly through miR-182/PCAT-1 participation.

### Enhanced expression of PCAT-1 triggers CAF infiltration via miR182/miR217 amplification

**Figure 5:**
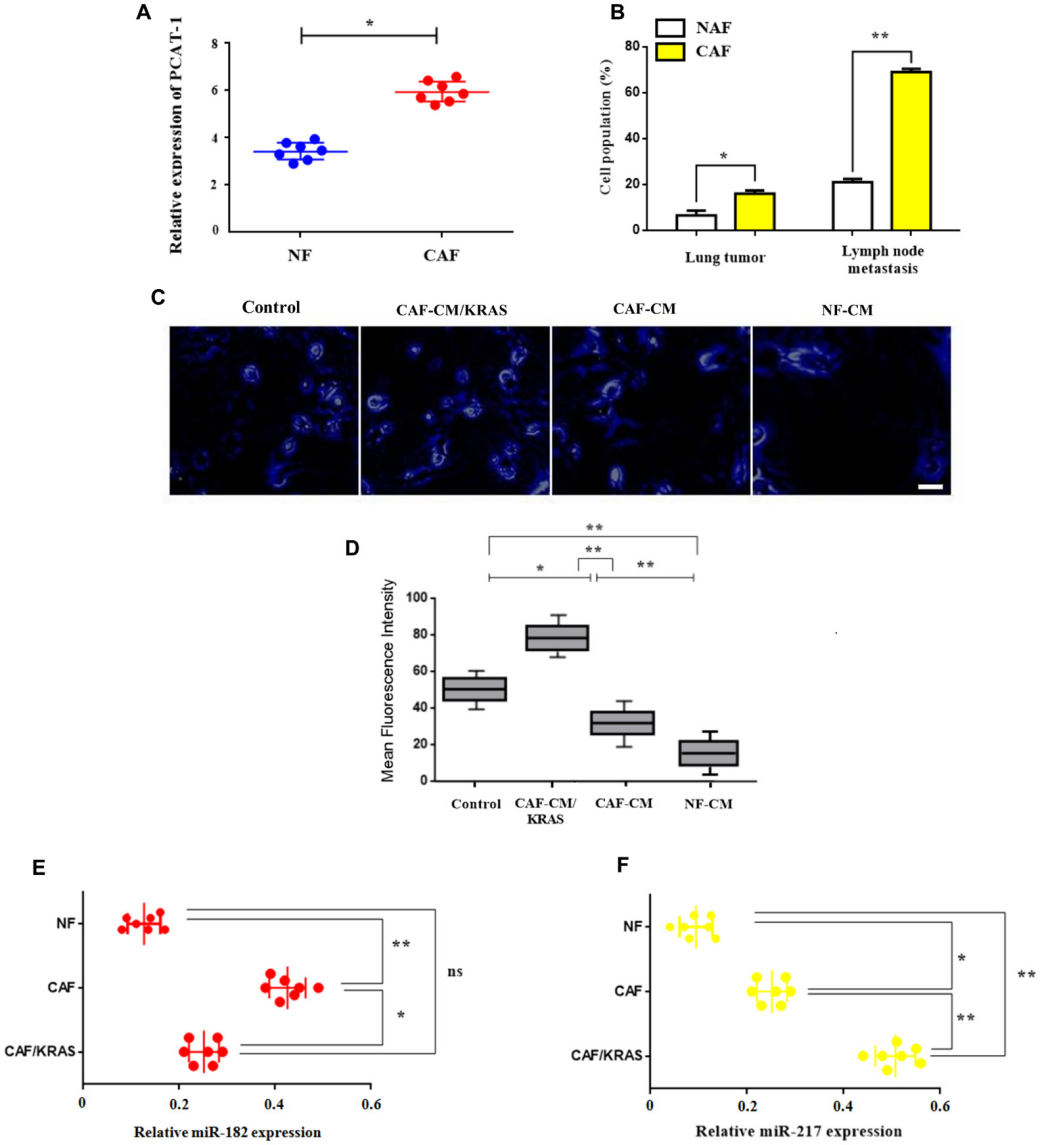
Enhanced expression of PCAT-1 triggers CAF infiltration via miR182/miR217 amplification. (**A**) mRNA expression levels of PCAT-1 in isolated CAFs or NAFs. (**B**) Percentage of cell population of CAFs/NAFs in primary lung tumor or LN metastatic tissues. The results represent the mean ± SD of three independent experiments. (**C**–**D**) Immunofluorescence analysis of PCAT-1 expression in H1975 cells (Magnification ×200). Cells were treated with various medium for 48 h. Images were captured using Carl Zeiss fluorescence confocal microscope. Data represent the mean ± SD of three independent experiments (^*^ *P* < 0.05; ^**^ *P* < 0.01). (**E**–**F**) Relative expression of miR-182/miR-217 in metastatic H1299 cells treated with CAF/CAF-Kras/NAF mediums derived from cancer patients in comparison with control (PBS). Data represent the mean ± SD of three independent experiments. Differences were considered statistically significant at *p* < 0.05. Statistically significant data are indicated by asterisks (^*^ *P* < 0.05, ^**^ *P* < 0.01).

To further comprehend the link between siPCAT-1 and Kras-dependent remodeling of lung microenvironment we decided to study the expression and systemic differentiation of fibroblast populations in primary and secondary tumors. Since fibroblasts are a heterogeneous population of differentiated fibrotic cells (CAFs, NAFs), their induction and infiltration as well as their correlation with disease progression remains unknown. For that reason we investigated the effect of fibroblast induction and infiltration in PCAT-1 expression. CAFs were isolated from lung tumor samples obtained from patients who had undergone surgical resection. Findings show an enrichment of PCAT-1 expression in CAF population within secondary tumors (Figure 5A). In contrast, PCAT-1 levels were reduced in NAF cells and their infiltration was lower in both primary and lymph node tumors (Figure 5B). Moreover, findings revealed that NAF to CAF differentiation and infiltration were increased dramatically and correlated with detection of metastatic lesions in lymph nodes (Figure 5D). To better comprehend the relationship between PCAT-1 expression and Kras-related CAF induction we employed conditioned medium (CM) derived from normal fibroblasts (NF) and CAFs/Kras (MT) patients. Data analysis depict an upregulation of PCAT-1 expression in CAF and CAF/Kras- treated cells compared to NF-CM group (Figure 5C–5D), indicating the essential role of Kras-dependent CAF differentiation in PCAT-1 induction. In addition, immunosuppressive miR-182/miR-217 levels were significantly increased in CAF and CAF/Kras-treated cells respectively (Figure 5E–5F).

### Exosomal PCAT-1 promotes tumor growth and guides lymph node metastasis *in vivo*

**Figure 6:**
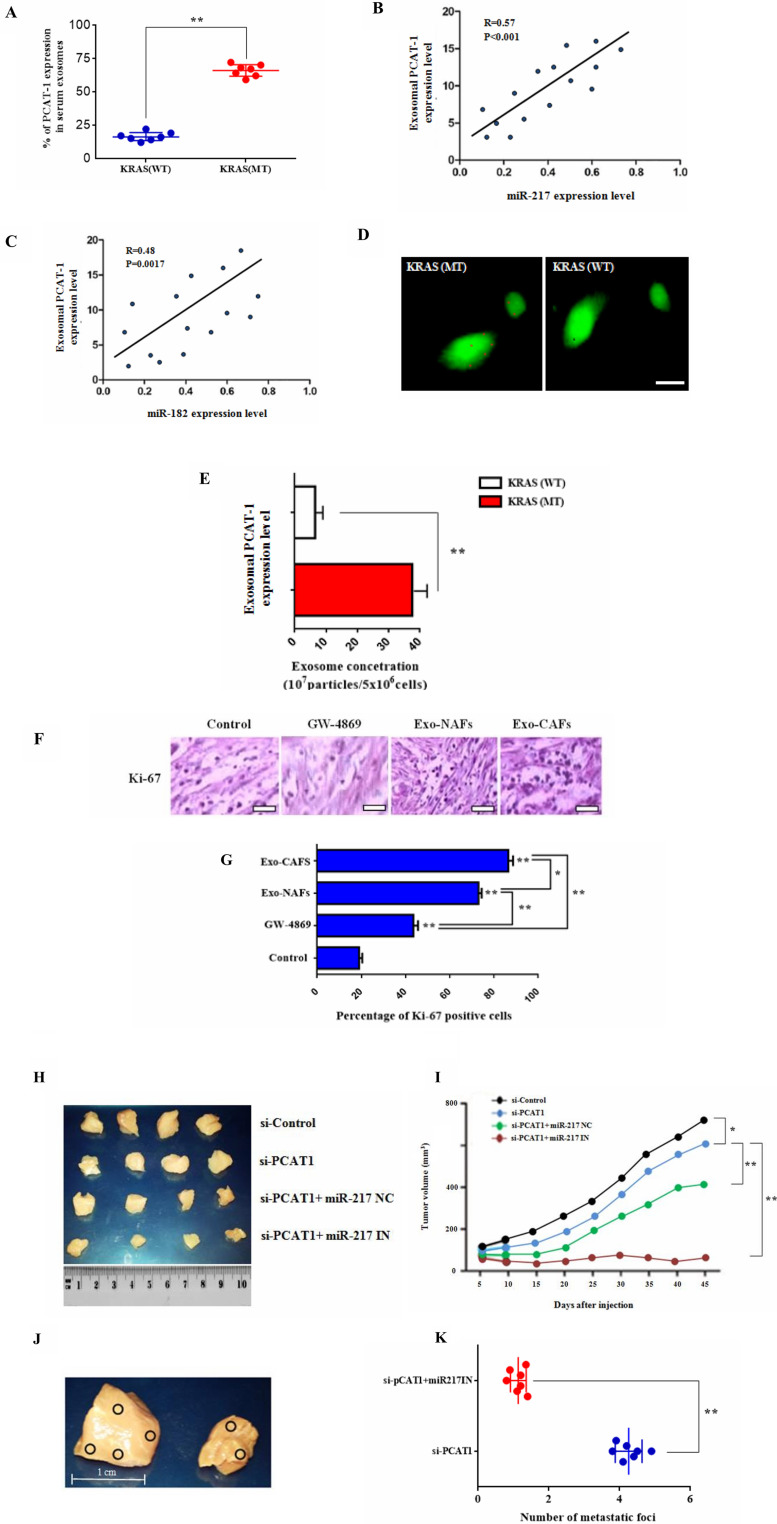
Exosomal PCAT-1 promotes tumor growth and guides lymph node metastasis *in vivo*. (**A**) Levels of PCAT-1 in serum exosomes were measured using RT-PCR. (**B**–**C**) Graph showing positive correlation between the miR-182/miR-217 ratio and exosomal PCAT-1 levels. Degree of correlation was higher in miR-217 in comparison with miR-182 (R = 0.57 vs R = 0.48) (**D**) Uptake of PKH67-labeled exosomes by LC/DR cells. Confocal microscopy image of PKH67-labeled cells (green) and exosomes (red). Images were captured using Carl Zeiss fluorescence confocal microscope. (**E**) Exosomal concentration of Kras (WT/MT) cells. Data represent the mean ± SD of three independent experiments (^*^ *P* < 0.05; ^**^ *P* < 0.01). (**F**) Immunohistochemical staining for Ki-67 in lung biopsies from control subjects and biopsies treated with exosomes and GW-4869. Sections of human lung biopsies were obtained from patients with lung cancer and following Kras/exosome inhibition treatment (50 μΜ, 1 h). Human Ki-67, was detected using anti-human monoclonal antibody. Staining with mouse IgG1 isotype was used as the negative control. (**G**) Quantification of immunohistochemistry by the intensity of the positive immunosignals in the tissue sections are showed in histograms (^*^ *P* < 0.05, ^**^ *P* < 0.01). Images were obtained through a Carl Zeiss microscope using image analysis software (Scale bar, 50 μm). Stained tumor cells are shown at a final magnification (×400). (**H**) Representative photographs of excised tumors from mice after treatment with siPCAT-1 and miR-217 inhibitor. (**I**) Tumor growth curve of mice treated in the different groups. Survival rates of tumor-bearing mice after a 60-day tumor challenge in each group. Data were given as the mean ± SD (*n* = 6) (^*^ *P* < 0.05, ^**^ *P* < 0.01). (**J**) *In vivo* metastatic analysis of lymph node metastasis from siPCAT-1 and siPCAT-1/miR-217 IN-treated groups. Images showed representative lymph node metastatic foci highlighted in yellow from different groups. (**K**) Statistical analysis of the number of metastatic foci of each group. Data represent the mean ± SD of three independent experiments. Differences were considered statistically significant at *p* < 0.05. Statistically significant data are indicated by asterisks (^*^ *P* < 0.05, ^**^ *P* < 0.01).

Latest studies depict that lncRNA expression is vastly regulated by exosomes present in lung tumor microenvironment, that create an immunosuppressive tumor promoting status [39, 40]. To investigate this hypothesis, we quantified PCAT-1 expression in exosomes, extracted from serum of LC patients (Supplementary Figure 3). As shown in Figure 6A, exosomal PCAT-1 is highly expressed in Kras (MT) patients in comparison with Kras (WT) individuals (Figure 6A). Similarly, a positive correlation was observed between PCAT-1 expression and miR182/miR-217 respectively (Figure 6B–6C). However, the degree of correlation was higher in miR-217 in comparison with miR-182 (R = 0.57 vs R = 0.48). To better understand this mechanism, we performed exosome characterization by electron microscopy analysis (Figure 6D). The results showed that exosomal PCAT-1 concentration is highly present in Kras (MT) group than in serum exosomes derived from Kras (WT) patients (Figure 6E). To further confirm the expression of exosomal PCAT-1 in tumor stroma, we isolated CAFs and NFs from LC tissues and adjacent normal tissues. Both Exo-CAF/NAF treatment significantly enhanced expression of the cell proliferation marker Ki-67. Further treatment with exosome inhibitor GW-4868 could not rescue PCAT-1- related proliferation (Figure 6F–6G). To better assess the effect of PCAT-1 *in vivo*, we tested the anti-tumor mechanism of PCAT inhibition in mouse model. Tumor examination analysis results revealed a significant decrease in the lung tumor volume of siPCAT-1-treated mice compared to vehicle treated mice (Figure 6H–6I). Further, co-treatment with siPCAT-1/miR-217 IN-treated cells, reduced significantly tumor growth in mice in comparison with siPCAT alone, siPCAT-1/miR-217 NC or control (PBS) group (Figure 6I). Next, to determine the effects of PCAT-1 inhibition on secondary metastasis, an *in vivo* metastasis assay was employed. Findings reveal, an increased number of lymph node metastatic foci in siPCAT-1/miR-217 IN group compared with siPCAT-1-treated mice (Figure 6J–6K) Furthermore, following treatment GW-4869- and GW-4869/siKras -treated mice exhibited reduced tumor formation, in comparison with control/siPCAT-1 groups (Figure 6J–6K). Data reveal that mice administered with siPAT-1 cells show a slightly delayed tumor progression accompanied by low level survival rate (data not shown). Mice injected with siPCAT-1 alone had a significantly shorter median overall survival after tumor implantation (32 days) than mice injected with the siPCAT-1/miR-217 IN (median overall survival, 48 days). These results confirm that blockage of PCAT-1/miR-217 pathway suppresses lung tumor growth along with metastatic potential and prolongs survival in nude mice.

## DISCUSSION

Emerging evidence reveal that tumor-induced immunosuppression in metastatic lung carcinoma mainly depends on the interactions of noncoding RNAs, including miRNAs and lncRNAs with tumor stroma and immune cells inside the tumor microenvironment [41, 42]. Recently NKILA, an NF-κB-interacting long noncoding RNA (lncRNA) was shown to promote tumor immune evasion by sensitizing T cells to activation-induced cell death and increasing CTL infiltration [43]. Similarly lncRNA SATB2-AS1 inhibits tumor metastasis and affects the tumor immune cell microenvironment by regulating SATB2 and increasing T_H1_-type chemokines expression and immune cell density [44]. Likewise, our findings reveal PCAT-1 to regulate Kras-related lung chemoresistance through increased expression of the immunosuppressive micrornas miR-182/miR217 in lung tissues, thus promoting a pre-metastatic niche formation and a subsequent increase in lung metastatic burden (Supplementary Figure 4). However, lncRNAs can also alter the metabolic profile of stroma cells and trigger tumor cell remodeling [45]. For example, LncRNA EPB41L4A-AS1 can regulate glycolysis and glutaminolysis by mediating nucleolar translocation of HDAC2. Specifically, it functions as a repressor of the Warburg effect and plays important roles in metabolic reprogramming of cancer cells [46]. Consistent with this, lncRNA ANRIL is up-regulated in nasopharyngeal carcinoma and promotes cancer progression via reprogramming cell glucose metabolism and inducing side-population stem-like cancer cells [47]. In many cases, lncRNAs are contained in exosomes and released by them inside the tumor stroma, mediating rapid immune suppression or chemoresistance [48]. Likewise exosome-transmitted lncARSR can promote sunitinib resistance by binding miR-34/miR-449 to facilitate AXL and c-MET expression in renal cancer, acting as a competing endogenous RNA [49]. Similarly, tumor-derived exosomal lnc-Sox2ot promotes EMT and stemness by acting as a ceRNA in pancreatic ductal adenocarcinoma [50]. In agreement with this, our results showed that exosomal PCAT-1 contained in CAFs triggers a CD133/SOX2-related stem cell phenotype which promotes tumor growth and guides lymph node metastasis *in vivo*. New data show that certain lncRNAs can reprogram stromal cells which are associated with the development and maintenance of immunosuppression [51]. For instance, Lnc-CAF reprograms fibroblasts to promote the growth of oral squamous cell carcinoma via p62-dependent autophagy-lysosome degradation of IL-33 and a-SMA/Ki67 upregulation [52]. Furthermore, tumor-associated macrophages (TAMs) enhance the aerobic glycolysis and apoptotic resistance of breast cancer cells via the extracellular vesicle (EV) transmission of a myeloid-specific lncRNA, HIF-1α-stabilizing long noncoding RNA (HISLA) [53]. Due to this dual role in balancing stroma cell reprogramming and tumor microenvironment remodeling, lncRNAs can influence the characteristics of neoplasia by favoring heterogeneity, metastatic potency and tumor-related chemoresistance [54, 55]. In conclusion, our findings reveal for the first time the key role of lncRNA PCAT-1 in regulating Kras-related lung chemoresistance and its role in tumor stroma remodeling via immunosuppressive miR-182/miR217 expression. Aberrant expression of PCAT-1 in the tumor microenvironment triggers fibroblast differentiation which negative regulates p27/CDK6 by inducing G_0_/G_1_ cell cycle arrest and AMPK augmentation, contributing to a tumor-favoring metabolic status. Our findings highlight the crucial relationship between CAFs and PCAT-1 which establish a CD133/SOX2-related stem cell phenotype and promote cancer cell chemoresistance. Decoding these molecular mechanisms and their impact in chemotherapy induction is essential for introducing novel immune-based strategies to restore and maintain antitumor immunity in chemoresistant metastasis.

## MATERIALS AND METHODS

### Ethics statement

The study was approved by the Ethics Committee on human experimentation of the AHEPA Hospital, Medical School, Aristotle University of Thessaloniki and informed consent was obtained from each patient before the surgical procedure.

### Lung cancer tissue samples

Lung cancer tissues were obtained from AHEPA Hospital, Medical School, Aristotle University of Thessaloniki (Thessaloniki, Greece). Tissue samples were collected in the operating room immediately after surgery with non-tumor tissues sent to Pathology for diagnosis by a certified pathologist. For each patient, a frozen tumor sample (stored at 80^°^C) and a paraffin-embedded tissue specimen were available. A written consent was obtained from 96 lung cancer (NSCLC) patients, 37–88 years of age, prior to surgery, from each patient voluntarily involved in the usage of tissues solely for research purposes (Supplementary Table 1). Patients had read and understood the patient information document provided, and the aims and methods of this study had been fully explained to them. Patients involved had also given written informed consent to authors of this manuscript for publication of these data. The study protocol was approved by the Investigational Review Board and Oncology Council of the ‘AHEPA’ General Hospital, Aristotle University of Thessaloniki (Ref. Number 734/2017). Written informed consent was obtained from each patient before study enrollment.

### Cell culture

Human lung adenocarcinoma cells A549 were grown in DMEM, supplemented with 10% fetal bovine serum, 2 mM L-glutamine, 1 mM sodium pyruvate, 100 U/ml penicillin, and 100 μg/ml streptomycin (Biochrom AG, Berlin, Germany). The epithelial lung cancer H1975 cells were grown in RPMI-1640 (Invitrogen, Carlsbad, USA) and were supplemented with 10% heat-inactivated fetal calf serum (FCS). Both cell lines were grown to confluence and maintained in a humidified atmosphere at 37°C and 5% CO2.

### *Ex vivo* tissue culture

Lung tumor samples from consenting Kras (chemoresistant/chemonaive) cancer patients at the AHEPA Hospital Oncology unit were collected, transferred to research lab in cold media, and processed in a sterile tissue culture hood. The culture protocol is modified based on previous studies [56–58]. Briefly, the culture medium consists of Dulbecco’s modified Eagles’s medium supplemented with 5% fetal calf serum (FCS), 10 mM HEPES, 0.5 μg/ml Hydrocortisone, 1% MEM vitamins solution, 5 μg/ml insulin, 100 IU/ml penicillin, 100 μg/ml streptomycin, and 15 μg/ml gentamicin. The tissue is placed in a sterile glass dish where all necrotic and visible connective tissue is removed. Tissue samples are cut in 0.5 cm^3^ to 1.0 cm^3^ slices via a cryotome and placed in a 6-well tissue culture dish with 2 ml of growth media. The dish is placed in a 37°C, 5% CO_2_ incubator for 24 h. After 1 day in culture, the tissue slices were subjected to immunohistochemical analysis.

### Animals

Six weeks old male athymic nude were purchased from the Jackson laboratory (Bar Harbor, ME) and were housed under pathogen-free environment. To establish xenografts in mice, A549 (3 × 10^6^) cells transfected with pcDNA-NC/pcDNA-PCAT-1 or miR-217NC/miR-217IN were subcutaneously inoculated into the left flank of the nude mice. Tumor volumes were calculated using the mathematical equation length × width^2^ × 0.5. Tumor volume was determined every week for 4 weeks after cell injection. The animals were then sacrificed, and xenograft tumors were weighed. Xenograft tumor samples were formalin fixed, paraffin-embedded, and sectioned. Then tissue sections were deparaffinized, rehydrated, and incubated with anti- Ki-67 antibody. The mice were maintained at the Department of Medicine, Animal Resources Facility of the Aristotle University. All of the protocols were approved by the Aristotle University Ethics Committee (AUEC) in accordance with the NIH Guidelines for the Care and Use of Laboratory Mice.

### Immunohistochemistry

Biopsy lung tissue samples were immediately collected after surgical procedure. Then, tissues were treated with anti-human Ki-67 monoclonal antibody (Applied Biosystems, Foster City, CA). Staining with mouse IgG1 isotype was used as the negative control. Images were obtained through a Carl Zeiss microscope using image analysis software (Carl Zeiss, Berlin, Germany). The number of positive stained cells relative to the total number of cells in the tissue sections and the intensity of the positive immunosignals were quantified with Aperio ImageScope software (Vista, CA).

### Statistical analysis

The results are expressed as mean ± SD from at least three separate experiments performed in triplicate. The differences between groups were determined with a two-tailed Student’s *t-test* or ANOVA using GraphPad software. The results represent the mean ± SD of at least three independent experiments. Differences were considered statistically significant at *p* < 0.05. Statistically significant data are indicated by asterisks (^*^ *p* < 0.05, ^**^ *p* < 0.01).

## SUPPLEMENTARY MATERIALS



## Abbreviations

LncRNA: Long non-coding RNA
CAFs: cancer-associated fibroblasts
PCAT-1: Prostate cancer-associated transcript 1
CDK6: cell division protein kinase 6
FOXP3: forkhead box P3
AMPK: AMP-activated protein kinase
HIF-1A: hypoxia-inducible factor 1-alpha
SOX2: SRY (sex determining region Y)-box 2
a-SMA: alpha-smooth muscle actin
ALDH1: aldehyde dehydrogenase 1

## References

[1] Bray F , Ferlay J , Soerjomataram I , Siegel RL , Torre LA , Jemal A . Global cancer statistics 2018: GLOBOCAN estimates of incidence and mortality worldwide for 36 cancers in 185 countries. CA Cancer J Clin. 2018; 68:394–424. 10.3322/caac.21492. 30207593

[2] Wong MCS , Lao XQ , Ho KF , Goggins WB , Tse SLA . Incidence and mortality of lung cancer: global trends and association with socioeconomic status. Sci Rep. 2017; 7:14300. 10.1038/s41598-017-14513-7. 29085026 PMC5662733

[3] Hirsch FR , Hirsch FR , Scagliotti GV , Mulshine JL , Kwon R , Curran WJ Jr , Wu YL , Paz-Ares L . Lung cancer: current therapies and new targeted treatments. Lancet. 2017; 389:299–311. 10.1016/S0140-6736(16)30958-8. 27574741

[4] Reck M , Rabe KF . Precision Diagnosis and Treatment for Advanced Non-Small-Cell Lung Cancer. N Engl J Med. 2017; 377:849–861. 10.1056/NEJMra1703413. 28854088

[5] Herbst RS , Morgensztern D , Boshoff C . The biology and management of non-small cell lung cancer. Nature. 2018; 553:446–454. 10.1038/nature25183. 29364287

[6] Peng WX , Koirala P , Mo YY . LncRNA-mediated regulation of cell signaling in cancer. Oncogene. 2017; 36:5661–5667. 10.1038/onc.2017.184. 28604750 PMC6450570

[7] Schmitt AM , Chang HY . Long Noncoding RNAs in Cancer Pathways. Cancer Cell. 2016; 29:452–463. 10.1016/j.ccell.2016.03.010. 27070700 PMC4831138

[8] Bhan A , Soleimani M , Mandal SS . Long Noncoding RNA and Cancer: A New Paradigm. Cancer Res. 2017; 77:3965–3981. 10.1158/0008-5472.CAN-16-2634. 28701486 PMC8330958

[9] Dhamija S , Diederichs S . From junk to master regulators of invasion: lncRNA functions in migration, EMT and metastasis. Int J Cancer. 2016; 139:269–280. 10.1002/ijc.30039. 26875870

[10] Fan C , Tang Y , Wang J , Xiong F , Guo C , Wang Y , Zhang S , Gong Z , Wei F , Yang L , He Y , Zhou M , Li X , et al. Role of long non-coding RNAs in glucose metabolism in cancer. Mol Cancer. 2017; 16:130. 10.1186/s12943-017-0699-3. 28738810 PMC5525357

[11] Huarte M . The emerging role of lncRNAs in cancer. Nat Med. 2015; 21:1253–1261. 10.1038/nm.3981. 26540387

[12] Kopp F , Mendell JT . Functional Classification and Experimental Dissection of Long Noncoding RNAs. Cell. 2018; 172:393–407. 10.1016/j.cell.2018.01.011. 29373828 PMC5978744

[13] Alvarez-Dominguez JR , Lodish HF . Emerging mechanisms of long noncoding RNA function during normal and malignant hematopoiesis. Blood. 2017; 130:1965–1975. 10.1182/blood-2017-06-788695. 28928124 PMC5669209

[14] Cao MX , Jiang YP , Tang YL , Liang XH . The crosstalk between lncRNA and microRNA in cancer metastasis: orchestrating the epithelial-mesenchymal plasticity. Oncotarget. 2017; 8:12472–12483. 10.18632/oncotarget.13957. 27992370 PMC5355358

[15] Adams BD , Parsons C , Walker L , Zhang WC , Slack FJ . Targeting noncoding RNAs in disease. J Clin Invest. 2017; 127:761–771. 10.1172/JCI84424. 28248199 PMC5330746

[16] Tang Y , Wang J , Lian Y , Fan C , Zhang P , Wu Y , Li X , Xiong F , Li X , Li G , Xiong W , Zeng Z . Linking long non-coding RNAs and SWI/SNF complexes to chromatin remodeling in cancer. Mol Cancer. 2017; 16:42. 10.1186/s12943-017-0612-0. 28212646 PMC5316185

[17] Yu B , Wang S . Angio-LncRs: LncRNAs that regulate angiogenesis and vascular disease. Theranostics. 2018; 8:3654–3675. 10.7150/thno.26024. 30026873 PMC6037039

[18] Chen X , Song E . Turning foes to friends: targeting cancer-associated fibroblasts. Nat Rev Drug Discov. 2019; 18:99–115. 10.1038/s41573-018-0004-1. 30470818

[19] Kalluri R . The biology and function of fibroblasts in cancer. Nat Rev Cancer. 2016; 16:582–598. 10.1038/nrc.2016.73. 27550820

[20] Yang X , Li Y , Zou L , Zhu Z . Role of Exosomes in Crosstalk Between Cancer-Associated Fibroblasts and Cancer Cells. Front Oncol. 2019; 9:356. 10.3389/fonc.2019.00356. 31131261 PMC6510008

[21] Fan Q , Yang L , Zhang X , Peng X , Wei S , Su D , Zhai Z , Hua X , Li H . The emerging role of exosome-derived non-coding RNAs in cancer biology. Cancer Lett. 2018; 414:107–115. 10.1016/j.canlet.2017.10.040. 29107112

[22] Kenkel JA , Tseng WW , Davidson MG , Tolentino LL , Choi O , Bhattacharya N , Seeley ES , Winer DA , Reticker-Flynn NE , Engleman EG . An Immunosuppressive Dendritic Cell Subset Accumulates at Secondary Sites and Promotes Metastasis in Pancreatic Cancer. Cancer Res. 2017; 77:4158–4170. 10.1158/0008-5472.CAN-16-2212. 28611041 PMC5550516

[23] Hill BS , Pelagalli A , Passaro N , Zannetti A . Tumor-educated mesenchymal stem cells promote pro-metastatic phenotype. Oncotarget. 2017; 8:73296–73311. 10.18632/oncotarget.20265. 29069870 PMC5641213

[24] Looi CK , Chung FF , Leong CO , Wong SF , Rosli R , Mai CW . Therapeutic challenges and current immunomodulatory strategies in targeting the immunosuppressive pancreatic tumor microenvironment. J Exp Clin Cancer Res. 2019; 38:162. 10.1186/s13046-019-1153-8. 30987642 PMC6463646

[25] Yang F , Ning Z , Ma L , Liu W , Shao C , Shu Y , Shen H . Exosomal miRNAs and miRNA dysregulation in cancer-associated fibroblasts. Mol Cancer. 2017; 16:148. 10.1186/s12943-017-0718-4. 28851377 PMC5576273

[26] Zhuo C , Xu Y , Ying M , Li Q , Huang L , Li D , Cai S , Li B . FOXP3+ Tregs: heterogeneous phenotypes and conflicting impacts on survival outcomes in patients with colorectal cancer. Immunol Res. 2015; 61:338–347. 10.1007/s12026-014-8616-y. 25608795

[27] Eichmüller SB , Osen W , Mandelboim O , Seliger B . Immune Modulatory microRNAs Involved in Tumor Attack and Tumor Immune Escape. J Natl Cancer Inst. 2017; 109:djx034. 10.1093/jnci/djx034. 28383653

[28] Kohlhapp FJ , Mitra AK , Lengyel E , Peter ME . MicroRNAs as mediators and communicators between cancer cells and the tumor microenvironment. Oncogene. 2015; 34:5857–5868. 10.1038/onc.2015.89. 25867073 PMC4604012

[29] Bu L , Baba H , Yoshida N , Miyake K , Yasuda T , Uchihara T , Tan P , Ishimoto T . Biological heterogeneity and versatility of cancer-associated fibroblasts in the tumor microenvironment. Oncogene. 2019; 38:4887–4901. 10.1038/s41388-019-0765-y. 30816343

[30] Ahmed N , Escalona R , Leung D , Chan E , Kannourakis G . Tumor microenvironment and metabolic plasticity in cancer and cancer stem cells: Perspectives on metabolic and immune regulatory signatures in chemoresistant ovarian cancer stem cells. Semin Cancer Biol. 2018; 53:265–281. 10.1016/j.semcancer.2018.10.002. 30317036

[31] Plava J , Cihova M , Burikova M , Matuskova M , Kucerova L , Miklikova S . Recent advances in understanding tumor stroma-mediated chemoresistance in breast cancer. Mol Cancer. 2019; 18:67. 10.1186/s12943-019-0960-z. 30927930 PMC6441200

[32] Yang Z , Zhao S , Zhou X , Zhao H , Jiang X . PCAT-1: A pivotal oncogenic long non-coding RNA in human cancers. Biomed Pharmacother. 2019; 110:493–499. 10.1016/j.biopha.2018.12.014. 30530229

[33] Zhang J , Bu X , Wang H , Zhu Y , Geng Y , Nihira NT , Tan Y , Ci Y , Wu F , Dai X , Guo J , Huang YH , Fan C , et al. Cyclin D-CDK4 kinase destabilizes PD-L1 via cullin 3-SPOP to control cancer immune surveillance. Nature. 2018; 553:91–95. 10.1038/nature25015. 29160310 PMC5754234

[34] Micheletti R , Plaisance I , Abraham BJ , Sarre A , Ting CC , Alexanian M , Maric D , Maison D , Nemir M , Young RA , Schroen B , González A , Ounzain S , et al. The long noncoding RNA Wisper controls cardiac fibrosis and remodeling. Sci Transl Med. 2017; 9:eaai9118. 10.1126/scitranslmed.aai9118. 28637928 PMC5643582

[35] Bernardes de Jesus B , Marinho SP , Barros S , Sousa-Franco A , Alves-Vale C , Carvalho T , Carmo-Fonseca M . Silencing of the lncRNA Zeb2-NAT facilitates reprogramming of aged fibroblasts and safeguards stem cell pluripotency. Nat Commun. 2018; 9:94. 10.1038/s41467-017-01921-6. 29311544 PMC5758807

[36] Gopal K , Gupta N , Zhang H , Alshareef A , Alqahtani H , Bigras G , Lewis J , Douglas D , Kneteman N , Lavasanifar A , Lai R . Oxidative stress induces the acquisition of cancer stem-like phenotype in breast cancer detectable by using a Sox2 regulatory region-2 (SRR2) reporter. Oncotarget. 2016; 7:3111–3127. 10.18632/oncotarget.6630. 26683522 PMC4823094

[37] Zhang Y , Xu Y , Feng L , Li F , Sun Z , Wu T , Shi X , Li J , Li X . Comprehensive characterization of lncRNA-mRNA related ceRNA network across 12 major cancers. Oncotarget. 2016; 7:64148–64167. 10.18632/oncotarget.11637. 27580177 PMC5325432

[38] Tay Y , Rinn J , Pandolfi PP . The multilayered complexity of ceRNA crosstalk and competition. Nature. 2014; 505:344–352. 10.1038/nature12986. 24429633 PMC4113481

[39] Sun Z , Yang S , Zhou Q , Wang G , Song J , Li Z , Zhang Z , Xu J , Xia K , Chang Y , Liu J , Yuan W . Emerging role of exosome-derived long non-coding RNAs in tumor microenvironment. Mol Cancer. 2018; 17:82. 10.1186/s12943-018-0831-z. 29678180 PMC5909226

[40] Yousefi H , Maheronnaghsh M , Molaei F , Mashouri L , Reza Aref A , Momeny M , Alahari SK . Long noncoding RNAs and exosomal lncRNAs: classification, and mechanisms in breast cancer metastasis and drug resistance. Oncogene. 2020; 39:953–974. 10.1038/s41388-019-1040-y. 31601996

[41] Robbins PD , Morelli AE . Regulation of immune responses by extracellular vesicles. Nat Rev Immunol. 2014; 14:195–208. 10.1038/nri3622. 24566916 PMC4350779

[42] Sullenger BA , Nair S . From the RNA world to the clinic. Science. 2016; 352:1417–1420. 10.1126/science.aad8709. 27313039 PMC6035743

[43] Huang D , Chen J , Yang L , Ouyang Q , Li J , Lao L , Zhao J , Liu J , Lu Y , Xing Y , Chen F , Su F , Yao H , et al. NKILA lncRNA promotes tumor immune evasion by sensitizing T cells to activation-induced cell death. Nat Immunol. 2018; 19:1112–1125. 10.1038/s41590-018-0207-y. 30224822

[44] Xu M , Xu X , Pan B , Chen X , Lin K , Zeng K , Liu X , Xu T , Sun L , Qin J , He B , Pan Y , Sun H , et al. LncRNA SATB2-AS1 inhibits tumor metastasis and affects the tumor immune cell microenvironment in colorectal cancer by regulating SATB2. Mol Cancer. 2019; 18:135. 10.1186/s12943-019-1063-6. 31492160 PMC6729021

[45] Peng D , Tanikawa T , Li W , Zhao L , Vatan L , Szeliga W , Wan S , Wei S , Wang Y , Liu Y , Staroslawska E , Szubstarski F , Rolinski J , et al. Myeloid-Derived Suppressor Cells Endow Stem-like Qualities to Breast Cancer Cells through IL6/ STAT3 and NO/NOTCH Cross-talk Signaling. Cancer Res. 2016; 76:3156–3165. 10.1158/0008-5472.CAN-15-2528. 27197152 PMC4891237

[46] Liao M , Liao W , Xu N , Li B , Liu F , Zhang S , Wang Y , Wang S , Zhu Y , Chen D , Xie W , Jiang Y , Cao L , et al. LncRNA EPB41L4A-AS1 regulates glycolysis and glutaminolysis by mediating nucleolar translocation of HDAC2. EBioMedicine. 2019; 41:200–213. 10.1016/j.ebiom.2019.01.035. 30796006 PMC6444057

[47] Zou ZW , Ma C , Medoro L , Chen L , Wang B , Gupta R , Liu T , Yang XZ , Chen TT , Wang RZ , Zhang WJ , Li PD . LncRNA ANRIL is up-regulated in nasopharyngeal carcinoma and promotes the cancer progression via increasing proliferation, reprograming cell glucose metabolism and inducing side-population stem-like cancer cells. Oncotarget. 2016; 7:61741–61754. 10.18632/oncotarget.11437. 27557514 PMC5308687

[48] Xu R , Rai A , Chen M , Suwakulsiri W , Greening DW , Simpson RJ . Extracellular vesicles in cancer-implications for future improvements in cancer care. Nat Rev Clin Oncol. 2018; 15:617–638. 10.1038/s41571-018-0036-9. 29795272

[49] Qu L , Ding J , Chen C , Wu ZJ , Liu B , Gao Y , Chen W , Liu F , Sun W , Li XF , Wang X , Wang Y , Xu ZY , et al. Exosome-Transmitted lncARSR Promotes Sunitinib Resistance in Renal Cancer by Acting as a Competing Endogenous RNA. Cancer Cell. 2016; 29:653–668. 10.1016/j.ccell.2016.03.004. 27117758

[50] Li Z , Jiang P , Li J , Peng M , Zhao X , Zhang X , Chen K , Zhang Y , Liu H , Gan L , Bi H , Zhen P , Zhu J , et al. Tumor-derived exosomal lnc-Sox2ot promotes EMT and stemness by acting as a ceRNA in pancreatic ductal adenocarcinoma. Oncogene. 2018; 37:3822–3838. 10.1038/s41388-018-0237-9. 29643475

[51] Galati D , Corazzelli G , De Filippi R , Pinto A . Dendritic cells in hematological malignancies. Crit Rev Oncol Hematol. 2016; 108:86–96. 10.1016/j.critrevonc.2016.10.006. 27931844

[52] Ding L , Ren J , Zhang D , Li Y , Huang X , Hu Q , Wang H , Song Y , Ni Y , Hou Y . A novel stromal lncRNA signature reprograms fibroblasts to promote the growth of oral squamous cell carcinoma via LncRNA-CAF/interleukin-33. Carcinogenesis. 2018; 39:397–406. 10.1093/carcin/bgy006. 29346528

[53] Chen F , Chen J , Yang L , Liu J , Zhang X , Zhang Y , Tu Q , Yin D , Lin D , Wong PP , Huang D , Xing Y , Zhao J , et al. Extracellular vesicle-packaged HIF-1α-stabilizing lncRNA from tumor-associated macrophages regulates aerobic glycolysis of breast cancer cells. Nat Cell Biol. 2019; 21:498–510. 10.1038/s41556-019-0299-0. 30936474

[54] Turley SJ , Cremasco V , Astarita JL . Immunological hallmarks of stromal cells in the tumor microenvironment. Nat Rev Immunol. 2015; 15:669–682. 10.1038/nri3902. 26471778

[55] Yarchoan M , Johnson BA , Lutz ER , Laheru DA , Jaffee EM . Targeting neoantigens to augment antitumor immunity. Nat Rev Cancer. 2017; 17:209–222. 10.1038/nrc.2016.154. 28233802 PMC5575801

[56] Domvri K , Petanidis S , Anestakis D , Porpodis K , Bai C , Zarogoulidis P , Freitag L , Hohenforst-Schmidt W , Katopodi T . Dual photothermal MDSCs-targeted immunotherapy inhibits lung immunosuppressive metastasis by enhancing T-cell recruitment. Nanoscale. 2020; 12:7051–7062. 10.1039/D0NR00080A. 32186564

[57] Petanidis S , Domvri K , Porpodis K , Anestakis D , Freitag L , Hohenforst-Schmidt W , Tsavlis D , Zarogoulidis K . Inhibition of Kras-derived exosomes downregulates immunosuppressive BACH2/GATA-3 expression via RIP-3 dependent necroptosis and miR-146/miR-210 modulation. Biomed Pharmacother. 2020; 122:109461. 10.1016/j.biopha.2019.109461. 31918262

[58] Anestakis D , Petanidis S , Domvri K , Tsavlis D , Zarogoulidis P , Katopodi T . Carboplatin chemoresistance is associated with CD11b+/Ly6C+ myeloid release and upregulation of TIGIT and LAG3/CD160 exhausted T cells. Mol Immunol. 2020; 118:99–109. 10.1016/j.molimm.2019.11.008. 31862674

