# Supplementary material for: Exosomal lncRNA PCAT-1 promotes Kras-associated chemoresistance via immunosuppressive miR-182/miR-217 signaling and p27/CDK6 regulation
Source: Oncotarget. 2020 Jul 21;11(29):2847–62. doi: 10.18632/oncotarget.27675 (PMC7381096; doi:10.18632/oncotarget.27675)
Supplement: Supplementary file 1 [file oncotarget-11-2847-s001.pdf]

Exosomal lncRNA PCAT-1 promotes Kras-associated chemoresistance via immunosuppressive miR-182/miR-217 signaling and p27/CDK6 regulation

SUPPLEMENTARY MATERIALS

Reagents

Carboplatin and K-Ras (G12C) Inhibitor was purchased from Sigma (Sigma, Germany, Europe). Polyclonal antibodies to phospho-AKT (Ser-473), phospho-AKT (Thr-308), Phospho-AMPKα (Thr172), phospho-p38 (Thr-180/Tyr-182), ERK1, p38, phospho-JNK1/2 (Thr-183/Tyr-185), and N-cadherin were obtained from Cell Signaling Technology (Beverly, MA). Monoclonal antibodies for p21, p27, SMARCE1, CDK4, ARID1A, CDK6, PKM2, RIP1, RIP3 were purchased from Santa Cruz Biotechnology (Santa Cruz, CA, USA). Rabbit polyclonal antibodies for BACH2, GATA3, BAX and PUMA were purchased from Abcam (Cambridge, UK). Dulbecco's modified eagle medium (DMEM) and fetal bovine serum (FBS) were obtained from Invitrogen (Invitrogen, Paisley, UK). DMSO (Dimethyl sulfoxide) and Tris-EDTA (Tris-Ethylene Diamine Tetraacetic Acid) were purchased from Applichem (Applichem, Darmstadt, Germany). DMEM-Hepes, PBS (Phosphate Buffer Saline), FBS (Fetal Bovine Serum), Trypsin–EDTA, and DMSO reagents were purchased from Invitrogen (Invitrogen, Darmstadt, Germany). The stock solution was filtered through a 0.22 μM syringe filter, then aliquoted and stored in the dark at room temperature.

Lung cancer tissue samples

Lung cancer tissues were obtained from AHEPA Hospital, Medical School, Aristotle University of Thessaloniki (Thessaloniki, Greece). Tissue samples were collected in the operating room immediately after surgery with non-tumor tissues sent to Pathology for diagnosis by a certified pathologist. For each patient, a frozen tumor sample (stored at 80°C) and a paraffin-embedded tissue specimen were available. A written consent was obtained from 96 lung cancer (NSCLC) patients, 37–88 years of age, prior to surgery, from each patient voluntarily involved in the usage of tissues solely for research purposes (Supplementary Table 1). Patients had read and understood the patient information document provided, and the aims and methods of this study had been fully explained to them. Patients involved had also given written informed consent to authors of this manuscript for publication of these data. The study methodologies were approved by the local ethics committee. The clinical investigation was conducted according to the guidelines expressed in the Declaration of Helsinki.

CAF culture

CAFs were isolated from metastatic lung cancer patients (Stages III-IV) who underwent lobectomy at the "G. Papanikolaou" General Hospital (GPGH), Aristotle University of Thessaloniki, and the use of specimens was approved by the Institutional Review Board of GPGH. NAFs were isolated from lung tissues from normal biopsies. All samples were cut in small pieces and digested with collagenase type I (2 mg/mL) and hyaluronidase (100 units/mL) in DMEM without FBS at 37°C for 8 h. After filtering the undigested tissues, the stromal fraction was centrifuged at 1400 rpm for 4 min. Finally, isolated cells were cultured in DMEM supplemented with 10% FBS, 1% penicillin and 1% streptomycin at 37°C in a 5% CO2 incubator.

siRNA transfection

Transfection of cells with siRNA of PCAT-1 was performed using Lipofectamine 2000 (Invitrogen, Berlin, Germany).The following primers were employed: siPCAT- 1A: 5′-GCAGAAACACCAAUGGAUATT-3′ and 5′- UAUCCAUUGGUGUUUCUGCTT-3′; siPCAT-1B, 5′- AUACAUAAGACCAUGGAAATT-3′and 5′-UUUCCAUGGUCUUAUGUAUTT-3′.The negative control (NC) RNA duplex for miRNAs miR-182/mir-217 inhibitors, (Genepharma, Shanghai, China) was non homologous to any human genome sequence.

RNA extraction and RT-PCR

Trizol reagent (Takara, Dalian, China) was used to isolate cells total RNAs. Serum RNA was extracted from a 400 μl volume of serum samples by RNA extraction kit (Life Technologies, US). The cDNA was obtained by the reverse transcription kit (Thermo Fisher Scientific, US) following 42°C for 60 min and 70°C for 5 min. All amplification procedures were ran on ABI 7500 detection

system (ABI, USA). The PCAT-1 primer sequences were as follows: F5′-GAGAGCTGACATAGGCACCC-3′ and R5′-TCTCCACTGGTGTTCATGGC-3′; GAPDH: F5′- TGATGACATCAAGAAGGTGGTGAAG-3′ and R5′-TCCTTGGAGGCCATGTGGGCCAT-3′. The real-time PCR reactions were repeated three times. Relative expression of PCAT-1 was normalized to GAPDH or U6 and was determined by qRT-PCR using the 2−ΔΔCt method.

Plasmid construction and transfection

The LncRNA PCAT-1 vectors, which include the empty vector pcDNA3.1 (Vector), the PCAT-1 overexpressing vector pcDNA3.1-PCAT-1 (pcDNA-PCAT-1), and small interfering RNAs against PCAT-1 (siPCAT-1) or its scramble negative siRNA (sh- NC) were obtained from Genepharma (Shanghai, China). miR-con, miR-128 and anti- miR-128 were obtained from Sangon Biotech (Shanghai, China). Cell transfections were carried out using the Lipofectamine 2000 (Invitrogen, Carlsbad, CA, USA).

Quantitative real-time PCR (qRT-PCR)

Trizol reagent (TaKaRa, Tokyo, Japan) was used for total RNA extraction. Then, cDNA samples were obtained by reverse transcription using PrimeScript RT Reagent Kit (TaKaRa). The qRT-PCR was performed using SYBR green qRT-PCR assay. GAPDH was used as an internal control for PCAT-1 and U6 was used as an internal control for miRs-182/217. The 2−ΔΔCt method was used to analyze the data.

Tumor xenografts

BALB/c nude mice (4 weeks old, female) were raised in a sterile environment in Laboratory Animal Center of Nantong University. We achieved approval of the Animal Care Committee of Nantong University before our experiment. 3×10^6 A549 cells transfected with pcDNA-NC /pcDNA-PCAT-1 or miR-217NC/miR-217IN were subcutaneously inoculated into the left flank of the nude mice. Tumor volumes were calculated using the mathematical equation length × width^2 × 0.5. Tumor volume was determined every week for 4 weeks after cell injection. The animals were then sacrificed, and xenograft tumors were weighed. Xenograft tumor samples were formalin fixed, paraffin-embedded, and sectioned. Then tissue sections were deparaffinized, rehydrated, and incubated with anti- Ki-67 antibody.

Cell cycle analysis

NSCLC cells were harvested and fixed overnight in 70% ethanol at 4°C. The cells were then incubated with 1 mg/mL propidium iodide (Sigma Aldrich) and 10 mg/mL RNase at 37°C for 30 minutes. Finally, the cell cycle was determined via flow cytometry (BD Biosciences, San Diego, CA).

Transwell migration assay

Cell invasion was evaluated using a matrigel invasion chamber. The migration assay was conducted in a 24-well transwell cell culture apparatus fitted with multiporous polycarbonate membrane insert (8-µm pore size) (Corning). In brief, cells were collected and resuspended in serum free media at a density of 1 × 105 cells/ml. The top chamber of transwell was loaded with 100 µl of cell suspension and the lower chamber was filled with 0.5 ml of media supplemented with 10% FBS as a chemo attractant. After incubation at 37°C in 5% CO2 for 24 h, the filters were removed, rinsed two times with PBS, fixed with methanol and stained with 0.5% crystal violet for 20 min. Cells on the upper side of the filter were wiped off with cotton swabs. The cells that migrated on the lower side of the filter were determined by counting specified cross-sectional fields on the filter with a phase-contrast microscope. The experiments were performed in triplicates.

Transwell invasion assay

Transwell assay was performed to estimate the effects of miR-182/miR-217 inhibition or siPCAT-1 on the invasion of H1299/H1975 cells. Matrigel was diluted in serum free DMEM medium and 100 µl of the diluted matrigel was added into transwells containing 8 µm pores and dried out at 37°C for 4 h. Control cells were seeded into the upper chamber containing serum-free DMEM and 0.5 ml medium with 10% FBS was added to the lower chamber. Cells were given 48 h to invade through matrigel then transwells were removed from the 24-well plate and stained with 0.1% crystal violet. The non invaded cells on the top of the transwell were scraped off using a cotton swab. Cells were counted in five different fields with an inverted microscope.

Colony formation assay

Following transfection, the A549 cells and H1975 cells were counted and seeded in 12-well plates (in triplicate) at a density of 100 cells per well. The plates were incubated at 37°C and 5% CO2 in a humidified incubator. Fresh culture medium was replaced every 2 days. After 7 days of culture, the cells were stained with crystal violet, and the numbers of colonies were counted. The rate of colony formation was calculated using the following equation: colony formation rate = (number of colonies/number of seeded cells) × 100%.

AldeRed ALDH detection assay

The AldeRed with 588-A ALDH Detection Assay (scr-150, Millipore) was used, according to the

manufacturer's instructions. All samples were analysed using flow cytometry (BD Biosciences, San Diego, CA).

Exosome isolation, identification and quantification

For exosome isolation, cells were cultured in media supplemented with exosome- depleted FBS. FBS was depleted of bovine exosomes by passage through a 0.22-µm PVDF filter (Millipore) and ultracentrifugation at 130,000 *g* for 80 minutes. Exosome pellets were resuspended in 200 µL of phosphate-buffered saline (PBS) or medium with 1% exosome-depleted FBS. Circulating exosomes were isolated from human plasma with ExoQuick (System Bioscience), in line with the manufacturer's instructions. For identification by TEM analysis exosomes were fixed in 2% PFA (w/v) in 200 mM phosphate buffer (pH 7.4). Fixed exosomes were dropped onto a carbon-coated grid and left to dry at room temperature for 30 minutes. After washing in PBS, exosomes were fixed in 1% glutaraldehyde for 5 minutes, washed in water and stained with uranyl oxalate for 5 minutes. Samples were then embedded in 0.5% (w/v) uranyl acetate and 2.5% (w/v) methylcellulose and incubated on ice for 15 minutes. The excess liquid was then removed. The grid was dried at room temperature for 10 minutes and viewed at 120,000 magnification. LM10 nanoparticle characterization system (NanoSight) was used for real-time characterization and quantification of the vesicles in supernatant fractions and in samples from patients.

Total RNA and proteins in the exosomes and exosome-depleted supernatant were isolated for RT-PCR analysis and marker protein detection, respectively.

Exosome labeling

For exosome analysis experiments, purified exosomes from lung cancer patients were fluorescently labeled using PKH67 membrane dye (Sigma-Aldrich) following the manufacturer's instructions. Images were obtained using a Carl Zeiss confocal microscope (Carl Zeiss, Berlin, Germany).

Kras mutation detection

All patient tumor tissue specimens were snap-frozen and preserved in liquid nitrogen before use. Paraffin sections were then prepared and one section from each case was chosen for Hematoxylin-Eosin staining. An experienced pathologist analyzed the Hematoxylin-Eosin section to confirm the presence of tumor tissue. Subsequently, tissue samples from at least five serial sections were macrodissected to ensure that the specimens contained at least 80% tumor cells. DNA was isolated from these tissue samples using the Qiagen DNA extraction Kit (Qiagen, Berlin, Germany) according to manufacturer instructions. Subsequently, mutations in codon 12 of K-ras exon 1 were detected in these genomic DNA samples by PCR-based direct sequencing (Supplementary Table 2). The PCR primers for K-ras used were K-ras-Forward: 5′- AAGGCCTGCTGAAAGTGACTG-3′ and K-ras-Reverse: 5′- CTGGTGCAGGACCATTCTTCAG-3′. The PCR reaction was carried out in a total volume of 50 µl containing 150 ng of the extracted genomic DNA. PCR amplification conditions were as follows: initial denaturation at 94°C for 1 min, followed by 40 cycles of denaturation at 94°C for 30 s, annealing at 55°C for 30 s, and extension at 72°C for 30 s, with a final extension step at 72°C for 5 min in a TP-600 thermal cycler (TaKaRa). PCR products were then analyzed using 2.5% agarose gel electrophoresis and purified for further direct DNA sequencing through an ABI-3730XL DNA Analyzer (Applied Biosystems, Europe).

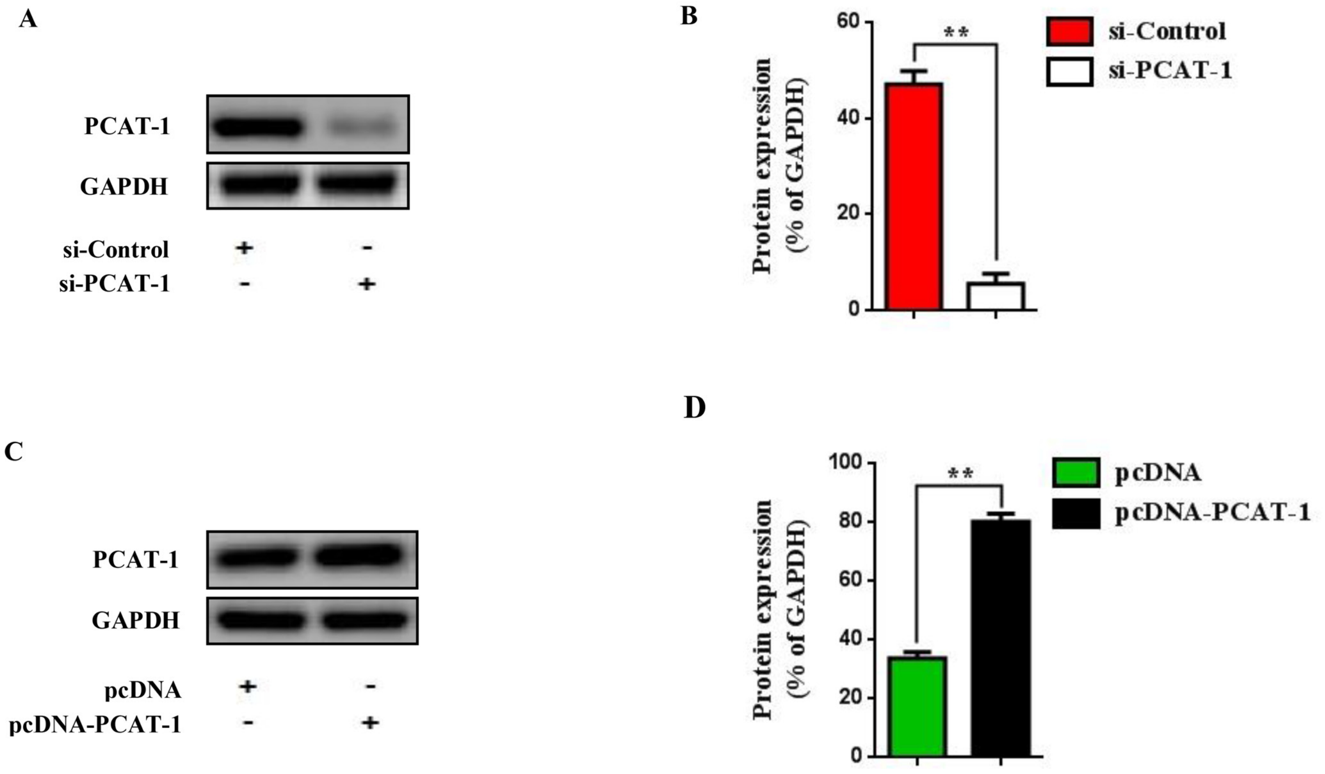

Supplementary Figure 1: (**A**–**B**) Knockdown of PCAT-1 in stably transfected A549 lung cancer cells. A549 cells were transfected with siRNA against PCAT-1 (4 μg) for 24 h. (**C**–**D**) Overexpression of PCAT-1 in H1975 cells transfected with pcDNA-PCAT-1/pcDNA or si-PCAT-1/si-Control vectors for a period of 72 h. Expression of PCAT-1 was examined by Western blot 48 h after transfection. Densitometric analysis of each protein level was calculated from the average of three experiments. Each value was expressed as the percentage of the measured protein to GAPDH level. Data represent the mean ± SD of three independent experiments ($^{*}P < 0.05$; $^{**}P < 0.01$).

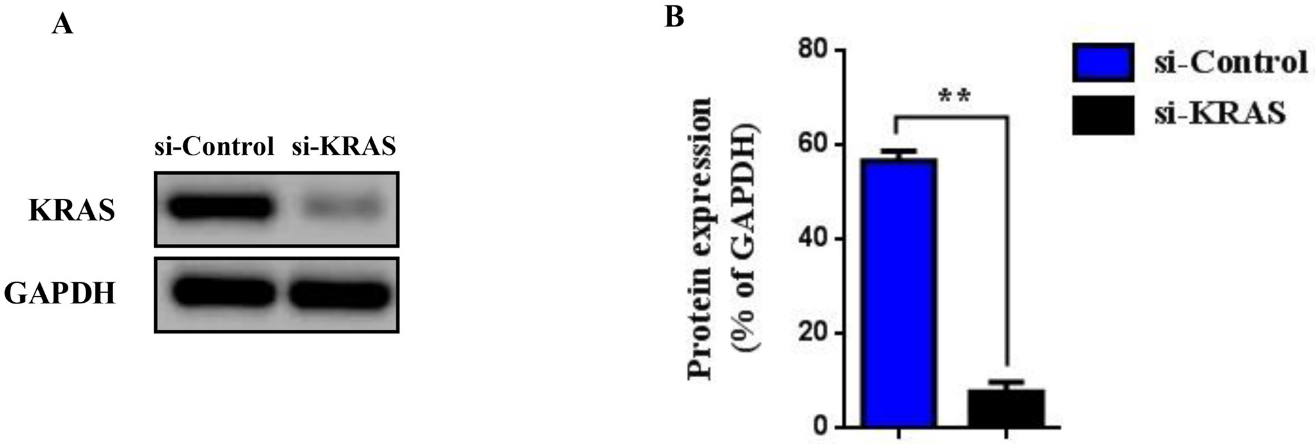

Supplementary Figure 2: Knockdown of KRAS in stably transfected H1975 lung cancer cells. (**A**) H1975 were transfected with siRNA targeting KRAS gene (si-KRAS) (4 µg) or scrambled siRNA (si-Control) for 24 h. Expression of KRAS was examined by Western blot 48 h after transfection. The intensity of the Western blot bands was quantified by scanning densitometry. (**B**) Densitometric analysis of each protein level was calculated from the average of three experiments. Each value was expressed as the percentage of measured protein to GAPDH level. Data are the mean ± SD of three independent experiments ($^{*}P < 0.05$, $^{**}P < 0.01$).

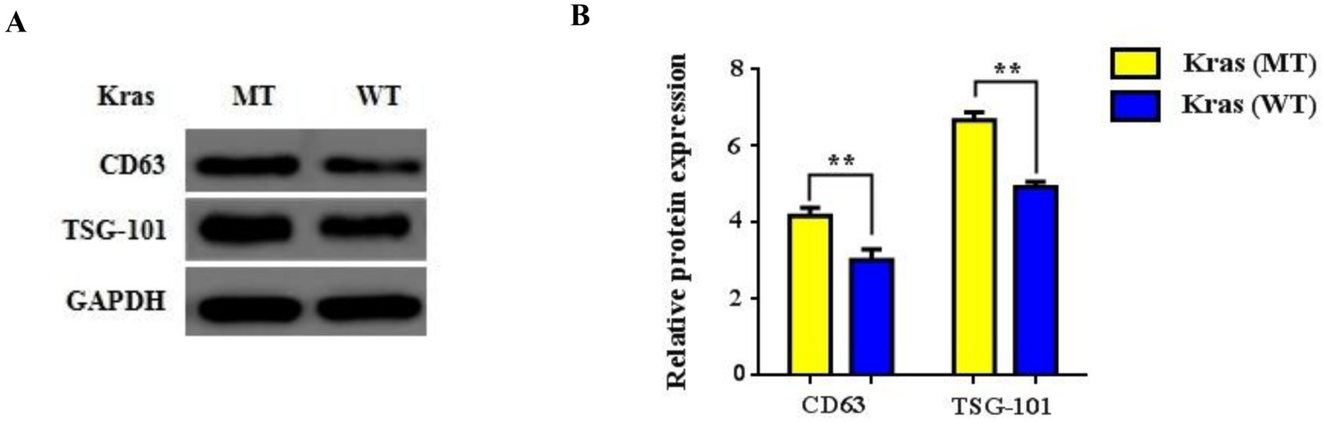

Supplementary Figure 3: (**A**) Exosome characterization from Kras (MT/WT) patients was performed using Western blot analysis from patient biopsies. Circulating exosomes were isolated from human plasma with ExoQuick (System Bioscience), in line with the manufacturer's instructions. (**B**) Immunoblotting of exosomal-related proteins CD63, and TSG-101. The intensity of the Western blot bands was quantified by scanning densitometry. Densitometric analysis of each protein level was calculated from the average of three experiments. Each value was expressed as the ratio of the measured protein to GAPDH level. Data are the mean ± SD of three independent experiments ($^{*}P < 0.05$, $^{**}P < 0.01$).

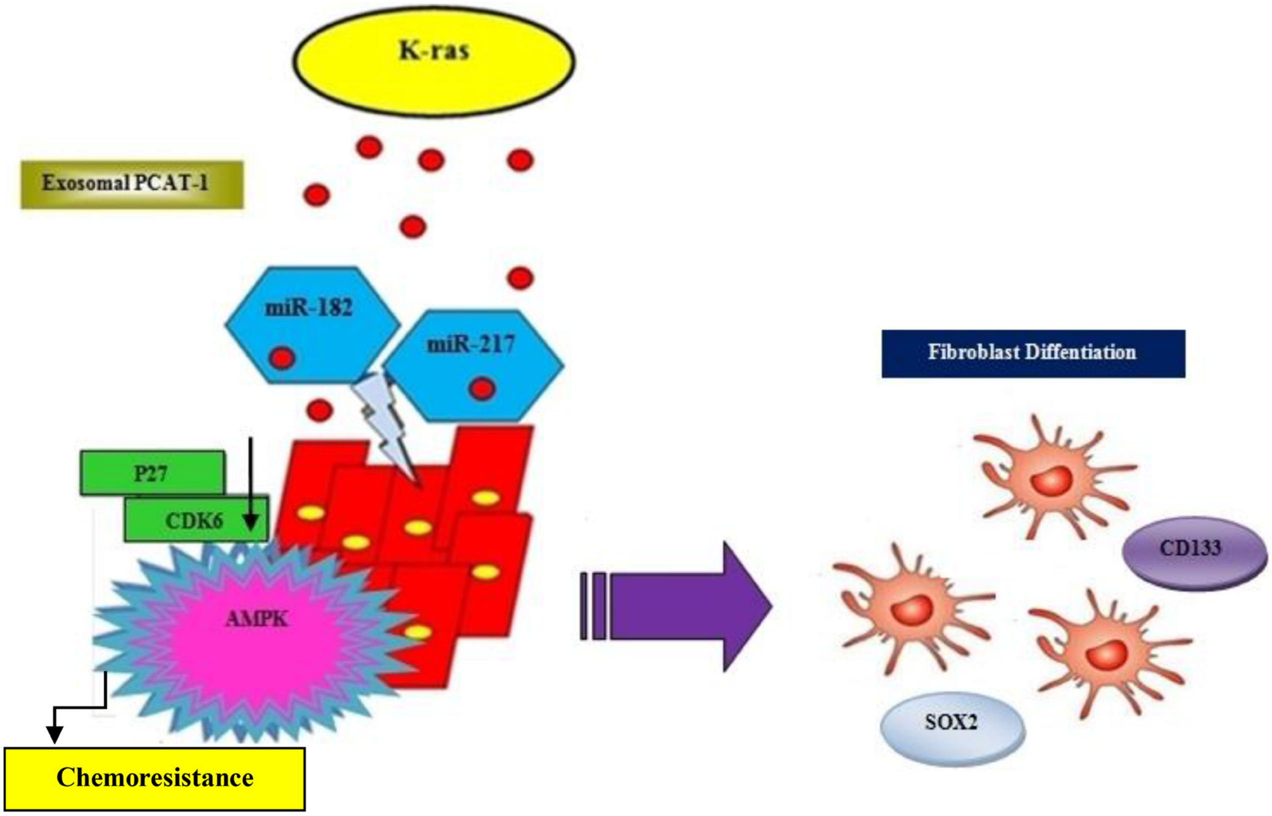

Supplementary Figure 4: Schematic diagram illustrating the mechanism of lnc-RNA PCAT-1 in Kras-related lung chemoresistance and its role in tumor stroma remodeling via immunosuppressive miR-182/miR217 expression and CD133/SOX2-mediated fibroblast differentiation.

Supplementary Table 1: Clinical characteristics of lung cancer patients

Clinical Characteristics	Mean Age	Sex		Histological Type		
		Male	Female	Squamous cell carcinoma	Adeno-carcinoma	Large cell carcinoma
Number of patients	67	56 (59)*	40 (41)	37 (39)	29 (30)	30 (31)

(*) Numbers in parenthesis indicate percentiles (%) in the total population of patients.

Supplementary Table 2: Correlation between KRAS mutation status in lung cancer patients

		KRAS codon 12 Mutation Status					
**Clinical Features**	***n***	**Gly12Cys (G12C)**	**Gly12Val (G12V)**	**Gly12Asp (G12D)**	**Gly12Arg (G12R)**	**Gly12Ala (G12A)**	**Gly12Ser (G12S)**
**Stage I**	24	10(42)	4(17)	4(17)	3(12)	1(4)	2(8)
**Stage II**	26	12(46)	5(19)	3(12)	3(12)	2(8)	1(4)
**Stage III**	42	17(40)	9(21)	5(12)	5(12)	3(7)	3(7)
**Stage IV**	32	19(60)	5(16)	3(9)	2(6)	2(6)	1(3)
**Chemoresistant**	51	28(55)	12(23)	4(8)	3(6)	1(2)	3(6)
**Chemosensitive**	45	11(24)	9(20)	8(18)	7(16)	6(13)	4(9)
**Metastatic**	47	32(68)	8(17)	3(6)	2(4)	1(2)	1(2)
**Non-metastatic**	49	12(24)	11(22)	8(16)	9(18)	5(10)	4(8)

Numbers in parenthesis indicate percentiles (%) in the total population of patients.

Supplementary Table 3: List of the gene primers used to perform mRNA analysis for the quantitative polymerase chain reaction (RT-PCR)

Gene	Primer
PCAT-1	Forward: 5′ - GAGAGCTGACATAGGCACCC -3′<br>Reverse: 5′- TCTCCACTGGTGTTCATGGC -3′
IL-13	Forward: 5′-TGAGGAGCTGAGCAACATACA -3′<br>Reverse: 5′-TGCGGTTACAGACACGGCC -3′
IL-33	Forward: 5′- GAAAGCGCAAGTCTTCAAAG -3′<br>Reverse: 5′- TGGGTAGGAGATGGAGATGC -3′
IFN-γ	Forward: 5′- GGCCATCAGCAACGGCGT-3′<br>Reverse: 5′- TGGGTTGTTGACTTGGC-3′
GATA-3	Forward: 5′- GTCTGCAGCCAGGAGAGC -3′<br>Reverse: 5′- ATGCATCAAACAACTGTCA -3′
GAPDH	Forward: 5′-ACCACAGTCCATGCCATCAC-3′<br>Reverse: 5′-TCCACCACCCTGTTGCTGT-3′
